# Center of Resistance for Maxillary Protraction in Patent and Fused Palatal-Suture Configurations: A Three-Dimensional Finite Element Study

**DOI:** 10.3390/bioengineering13080903

**Published:** 2026-08-10

**Authors:** Nattharin Wongsirichat, Yotsakorn Pratumwal, Aggasit Manosudprasit

**Affiliations:** 1 Department of Preventive Dentistry, Division of Orthodontics, Faculty of Dentistry, Khon Kaen University, Khon Kaen 40002, Thailand; nattharin.won@gmail.com; 2 National Metal and Materials Technology Center (MTEC), Pathum Thani 12120, Thailand; yotsakp@mtec.or.th

**Keywords:** Class III malocclusion, orthopedic force, biomechanics, nasomaxillary complex

## Abstract

Objective: To determine and compare the center of resistance (Cres) for maxillary protraction in fused and patent palatal suture models using three-dimensional finite element analysis. Study design: A three-dimensional craniofacial model was constructed from CBCT data of a 10-year-old female with maxillary hypoplasia. Models with fused and patent palatal sutures were created. Finite element analysis evaluated displacement patterns following anteroposterior forces (10 N) at different vertical positions (H1–H4) and superoinferior forces (10 N) at different sagittal positions (V1–V5). The force application site producing the most uniform displacement with minimal rotation was identified as the Cres. Results: In the fused palatal suture model, the Cres was located at H3.3 (infraorbital rim level, 15 mm superior to the line midway between the occlusal plane and the infraorbital rim.) for anteroposterior loading and at V3.6 (slightly distal to the lower border of the zygomatic process, 15 mm anterior to the posterior nasal spine) for superoinferior loading. In the patent palatal suture model, the Cres remained at similar levels but shifted laterally, requiring force application 20 mm lateral to the midpalatal suture to achieve translational movement. Force application closer to the midpalatal suture caused the maxillary segments to diverge anteriorly and converge posteriorly, whereas force application at the lateral maxillary rim produced the opposite rotational pattern. Conclusion: The Cres of the nasomaxillary complex was consistently located near the infraorbital rim under anteroposterior loading and slightly distal to the zygomatic process under superoinferior loading in both suture configurations. In patent sutures, optimal translational movement required bilateral force application 20 mm lateral to the midpalatal suture. These findings provide subject-specific biomechanical information that may assist in the design and evaluation of maxillary protraction force systems.

## 1. Introduction

Class III malocclusion has a relatively high prevalence in Southeast Asian populations. [1], primarily affecting the skeletal structure of the midface [2,3,4]. This condition is characterized by a protruding mandible and/or underdeveloped maxilla, where the mandible is positioned forward relative to the maxilla, resulting in an anterior crossbite relationship between the teeth. Clinically, Class III malocclusion is distinguished by several features, including anterior crossbite, reverse overjet, and functional shifts, where patients adopt a forward posture of the mandible to achieve a more favorable bite during functional activities [3,4,5].

The treatment approach for Class III malocclusion is mainly determined by the patient’s skeletal maturity status. Skeletally immature patients present multiple treatment options due to the potential for growth modification, including protraction facemask therapy, chin cup appliance therapy, and maxillary expansion when indicated. In contrast, adult patients require either orthodontic camouflage treatment, with or without dental extractions, or surgical intervention, specifically distraction osteogenesis or orthognathic surgery [5,6].

The circumaxillary and midpalatal suture complexes are critical anatomical structures that control craniofacial development and transmit orthopedic forces. Facial bone suture maturation patterns serve as essential biological indicators for maxillary protraction treatment planning. The timing of sutural fusion follows a predictable pattern: the pterygomaxillary suture typically fuses at approximately age 12, the zygomaticomaxillary suture at age 15, while the midpalatal suture mostly fuses at age 13 with complete fusion at age 18 [7,8,9,10]. The effectiveness of orthopedic intervention shows an age-dependent response, where younger patients demonstrate greater sutural flexibility and adaptive capacity, resulting in more favorable growth modifications.

When the craniofacial complex is loaded with forces in different directions, distinct displacement patterns and stress distributions occur at the nasal and maxillary buttresses, which are influenced by the center of resistance (Cres) of the nasal and maxillary complex. The Cres represents the theoretical point through which an applied force produces pure straight-line movement without rotational effects [11]. Understanding the precise location of the center of resistance has direct implications for orthodontic appliance design and treatment protocols. Current maxillary protraction appliances, including facemasks and bone-anchored maxillary protractors (BAMPs), often apply forces based on clinical observations rather than mechanically optimized force vectors. The suture opening status significantly influences the mechanical response to orthopedic forces, yet current clinical protocols rarely differentiate treatment approaches based on individual suture maturation status.

Finite element analysis (FEA) has evolved as a sophisticated computational method in contemporary craniofacial orthodontics. This analytical technique enables the simulation of mechanical force distribution patterns on bone and dental structures, facilitating measurement of predicted displacement vectors. The implementation of FEA methodology shows significant potential for optimizing maxillary protraction protocols through enhanced mechanical precision and therapeutic force control, potentially improving treatment effectiveness and clinical outcomes [12].

Previous studies examining the spatial coordinates of the nasomaxillary complex Cres have used diverse methods, including two-dimensional cephalometric analysis (typically localizing Cres near the posterior-superior cranial base) [13], and FEA (usually positioning Cres around the infraorbital rim or key ridge area) [14]. However, definitive anatomical localization remains incompletely defined due to the complex three-dimensional architecture of the nasomaxillary complex and methodological limitations of earlier studies. Notably, previous FEA studies mainly examined Cres in sagittal and vertical planes while neglecting transverse plane analysis, despite the inherently three-dimensional nature of the nasomaxillary complex. Furthermore, most prior research used adult skull models, which may not accurately represent the clinical scenario where facemask therapy is primarily implemented during adolescence with mixed dentition. Fundamental differences between adult and adolescent cranial shape include midpalatal suture fusion status, dental development stages, arch dimensions, cranial base configuration with active sphenooccipital synchondrosis growth, and maxillary shape changes with increased nasal and paranasal sinus development showing ongoing posterior-superior growth patterns causing characteristic downward and forward displacement [13]. Therefore, this study aimed to clarify the precise spatial coordination of Cres within the nasomaxillary complex through comprehensive three-dimensional FEA, incorporating both open and fused palatal suture shapes representative of growing craniofacial development.

## 2. Materials and Methods

### 2.1. Construction of Skull Model

Three-dimensional craniofacial morphology was assessed utilizing cone beam computed tomography (CBCT) imaging (WhiteFox cone-beam 3D system, operating parameters: 100 kV, 8 mA, field of view 200 mm × 170 mm, voxel size 0.3 mm) in a 10-year-old female subject presenting with maxillary hypoplasia (SNA ≤ 74°) [15] who was considered a candidate for orthopedic maxillary protraction. Patient selection was based on CBCT assessment. The SNA angle was reported to describe the sagittal maxillary position and the clinical indication for protraction therapy.

### 2.2. Software

DICOM (Digital Imaging and Communications in Medicine) datasets were processed using Mimics software 2023 (Materialise, Leuven, Belgium) for volumetric reconstruction and three-dimensional model generation. Image segmentation protocols used both manual and semi-automated algorithms to delineate osseous, dental, and soft tissue boundaries, with particular emphasis on the nasomaxillary complex and adjacent anatomical structures. SolidWorks 2017 (SolidWorks Corp., Waltham, MA, USA) was used to create skull computer aided design (CAD) models, and FEA was carried out using ANSYS Workbench 2021r2 (ANSYS Inc., Houston, TX, USA).

### 2.3. Mesh Generation, Material Properties and Boundary Condition

#### 2.3.1. Mesh Generation

**Figure 1:**
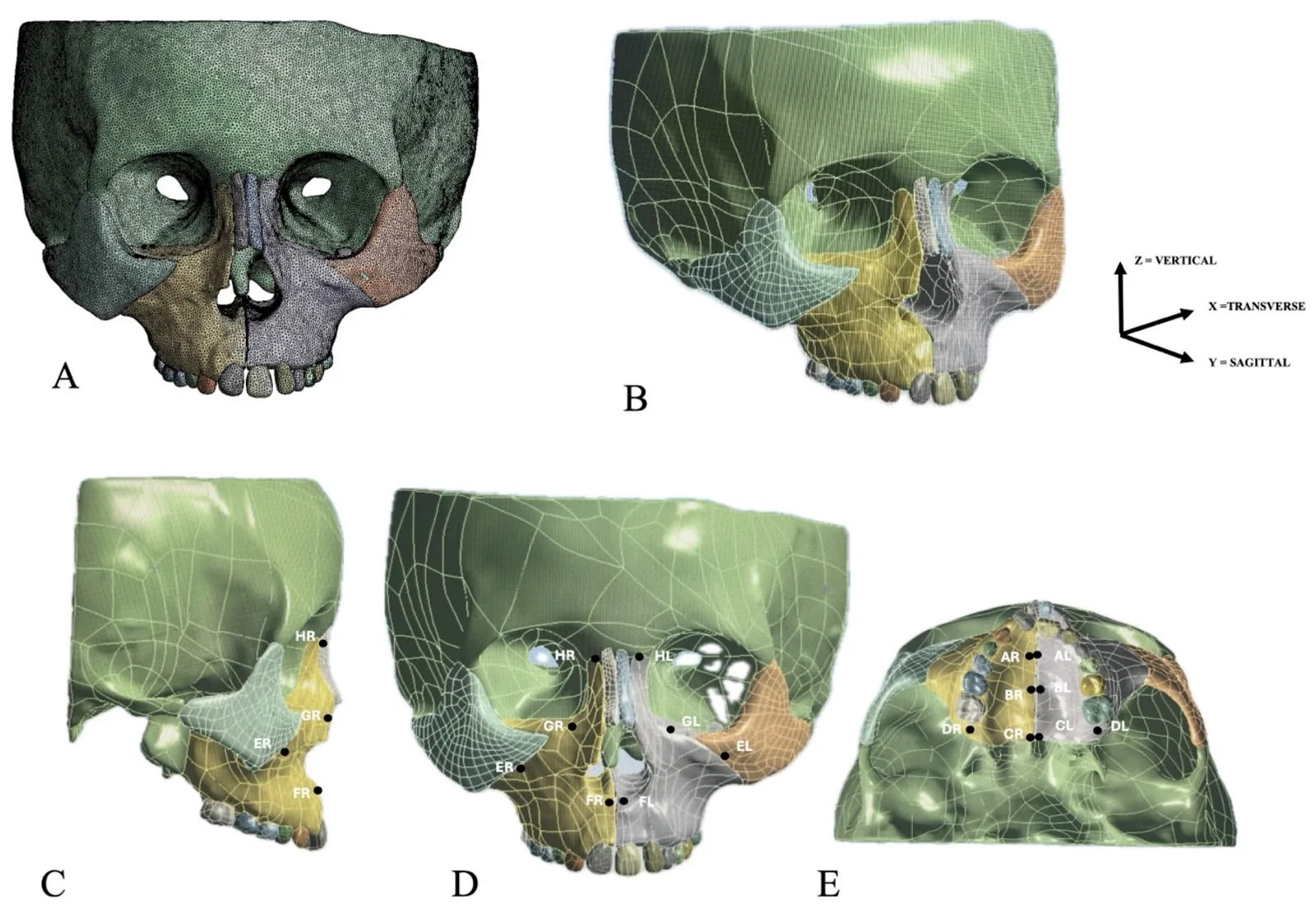
(**A**) Finite element skull model. (**B**) Coordinate system showing the X (transverse), Y (sagittal), and Z (vertical) axes. (**C**) Anatomical landmarks in the lateral view. (**D**) Anatomical landmarks in the frontal view. (**E**) Anatomical landmarks in the occlusal view.

The constructed three-dimensional finite element models included the cranial base, nasal bones, zygomatic complex, maxilla with dentition, and associated craniofacial sutures. All interfaces between the teeth, alveolar bone, and craniofacial bones were defined using surface-to-surface bonded contact, preventing relative sliding or separation during loading. The models were discretized using 4-node linear tetrahedral elements with a nominal element size of 1 mm, resulting in a final mesh comprising 968,156 nodes and 558,043 elements (Figure 1A). Mesh convergence analysis demonstrated numerical stability, with displacement differences of less than 1% between the final two mesh refinements, supporting the reliability of the computational results (Supplementary Table S1).

#### 2.3.2. Material Properties

Two computational models representing fused and patent midpalatal sutures were developed to investigate the influence of suture maturation on craniofacial biomechanics. In both configurations, the maxillary bone was assumed to be homogeneous and isotropic. The fused palatal suture model replicated a completely ossified suture by homogeneous integration of inter-sutural connections into a unified solid structure, creating a mechanically continuous maxillary complex representative of adult morphology. Conversely, the patent palatal suture model maintained discrete left and right maxillary segments with preserved inter-sutural space, with distinct elastic properties (Table 1) representing the more compliant pediatric suture. These model configurations reproduced the different mechanical behaviors and load-transfer characteristics of fused and patent sutures.

#### 2.3.3. Boundary Condition

Following previously established methodologies [14,16,17], the circumferential margin of the foramen magnum was constrained in all three translational directions (X, Y, and Z) to prevent rigid-body motion during computational analysis, while permitting rotational deformation of the craniofacial complex. Force application was divided into two loading conditions: an anteroposterior force applied along the vertical axis, and a superoinferior force applied along the sagittal axis.

### 2.4. Force-Loading Condition

#### 2.4.1. Anteroposterior Force-Loading Condition Along the Vertical Axis

##### Fused Palatal Suture Model

In the fused palatal suture model, simulated loading conditions involved bilaterally applied anteriorly directed forces of 5 N at the medial rim of the maxillary bones on the facial surface, based on the amount of extraoral forces for maxillary protraction commonly used in clinical practice [18,19], generating a total force of 10 N. A total force of 10 N (5 N per side) was applied because this magnitude has been reported in previous finite element studies of maxillary protraction and represents a clinically relevant orthopedic loading condition for evaluating displacement patterns of the nasomaxillary complex.

**Figure 2:**
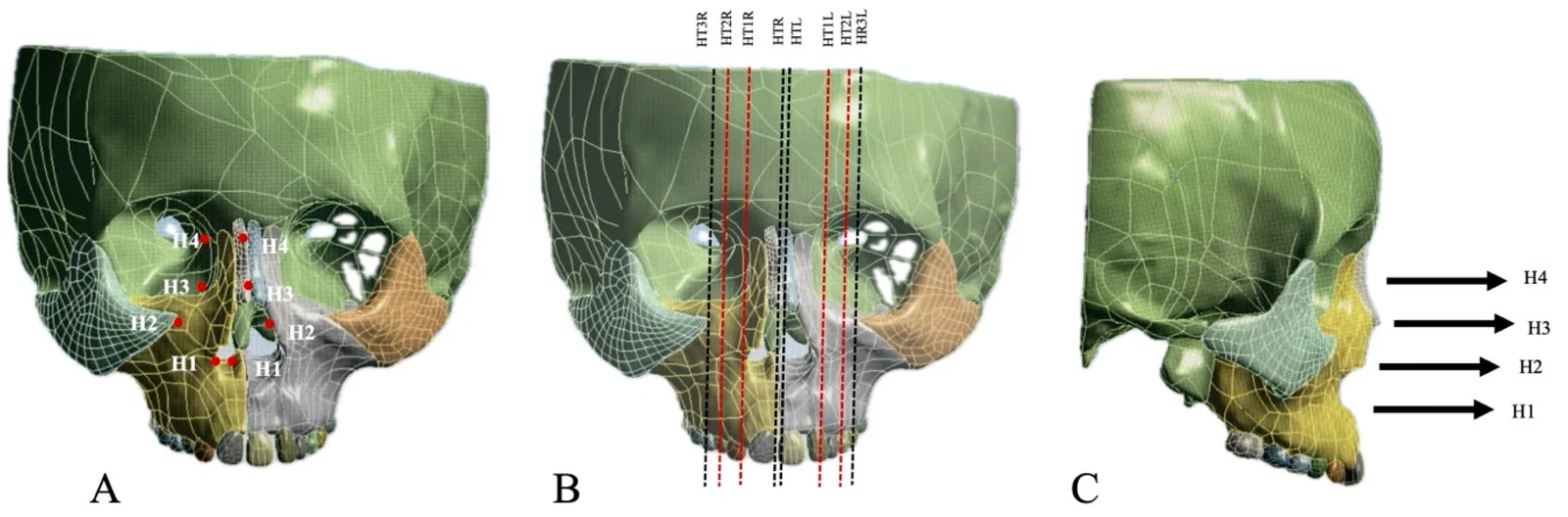
Point of loading for anteroposterior force application in frontal view (**A**), and lateral view (**C**) in fused palatal suture, in frontal view (**B**) in patent palatal suture model.

Four anteroposterior forces at vertical levels (H1–H4) were evaluated to determine the optimal location causing nearly pure translatory anterior displacement of the maxillary bone. Force levels were defined vertically as: H1 at the anterior nasal spine, H2 at midway between the occlusal and the infraorbital rim, H3 positioned 15 mm superior to H2, and H4 located 30 mm superior to H2 (Figure 2A,C).

##### Patent Palatal Suture Model

In the patent palatal suture model, with separated left and right maxillae, the force line from the fused palatal suture model was applied transversely at the medial rim of the maxillary bone (HT), laterally at 10 mm (HT1) and 20 mm (HT2) from the midline and 30 mm from the midline which is the lateral rim of the maxillary bone (HT3) as illustrated in Figure 2B.

#### 2.4.2. Superoinferior Force-Loading Condition Along the Sagittal Axis

##### Fused Palatal Suture Model

**Figure 3:**
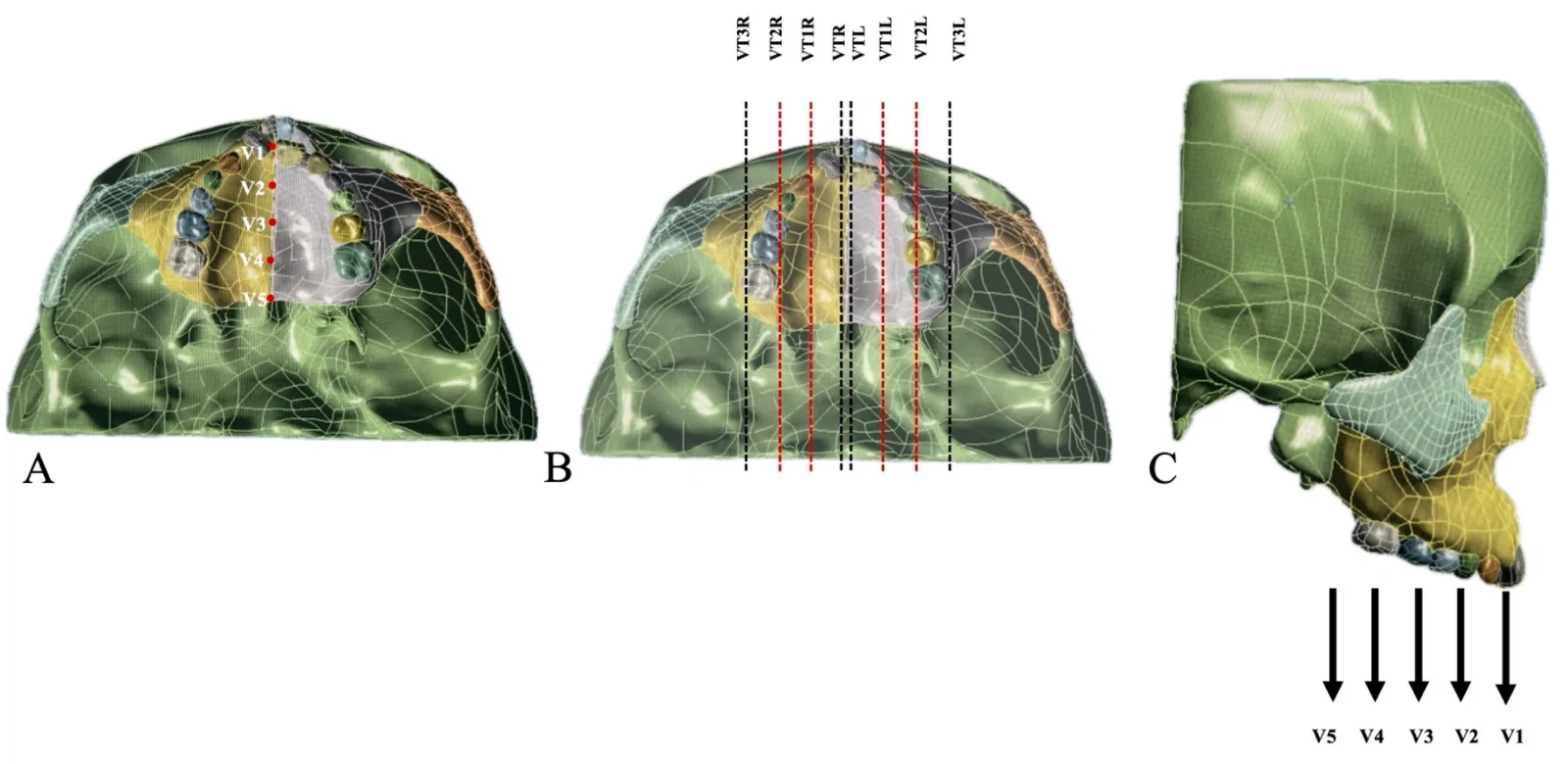
Point of loading for superoinferior force application in frontal view (**A**), and lateral view (**C**) in fused palatal suture, in frontal view (**B**) in patent palatal suture model.

In the sagittal axis analysis, bilateral superoinferior forces of 5 N were applied at the medial margins of the median palatine suture, generating a cumulative force magnitude of 10 N in the fused suture model. Five distinct force vectors (V1–V5) were systematically evaluated along the palatine sutural axis to determine the optimal spatial coordinates for achieving predominantly translatory displacement of the nasomaxillary complex vertically. The force vectors were spatially distributed in the sagittal direction relative to the posterior nasal spine (PNS) as follows: V1 at 40 mm anterior to PNS, V2 at 30 mm anterior to PNS, V3 at 20 mm anterior to PNS, V4 at 10 mm anterior to PNS, and V5 at PNS (Figure 3A,C).

##### Patent Palatal Suture Model

In the patent suture configuration, bilateral superoinferior forces of 5 N were applied to each maxillary segment at predetermined transverse distances from the midpalatal reference plane: at the parasagittal margins (VT), 10 mm (VT1), 20 mm (VT2) lateral to midline, and 30 mm from the midline which is the lateral rim of the maxillary bone (VT3), utilizing the established force vector from the fused model analysis, as depicted in Figure 3B.

### 2.5. Data Analysis

Eight anatomical landmarks, modified from established protocols by Tanne et al. (1995) and Lee et al. (2014) [14,20], were strategically positioned to quantify horizontal and vertical displacement patterns. The landmarks consisted of the incisive foramen (AR, AL), mid-palatal point (BR, BL), posterior nasal spine (CR, CL), maxillary tuberosity (DR, DL), zygomaticomaxillary suture (ER, EL), anterior nasal spine (FR, FL), infraorbital rim (GR, GL), and nasomaxillary suture (HR, HL) as demonstrated in Figure 1. These measurement points include the intermaxillary suture (connecting the bilateral maxillary bones anteriorly), the frontonasal suture (articulating the frontal and nasal bones), the frontozygomatic suture (joining the frontal and zygomatic bones), the zygomaticomaxillary suture (linking the zygomatic and maxillary bones), and the zygomaticotemporal suture (connecting the zygomatic and temporal bones). The mechanical properties of individual model components were quantitatively defined according to parameters presented in Table 1, with all constituent materials characterized as isotropic exhibiting linear elastic behavioral properties in accordance with established biomechanical modeling principles. [21,22] The mechanical response of the finite element models was evaluated using displacement components corresponding to the direction of the applied load. Under anteroposterior loading, displacement was analyzed along the sagittal (X) axis, whereas under superoinferior loading, displacement was analyzed along the vertical (Z) axis. Rotational motion was not quantified directly; instead, rotational tendency was inferred from differences in displacement among the predefined anatomical landmarks. The loading position that produced the most uniform translational displacement pattern across all anatomical landmarks was identified as the estimated center of resistance.

## 3. Result

### 3.1. Determining the Vertical Location of Center of Resistance Using Anteroposterior Forces

#### 3.1.1. Fused Palatal Suture Model

**Figure 4:**
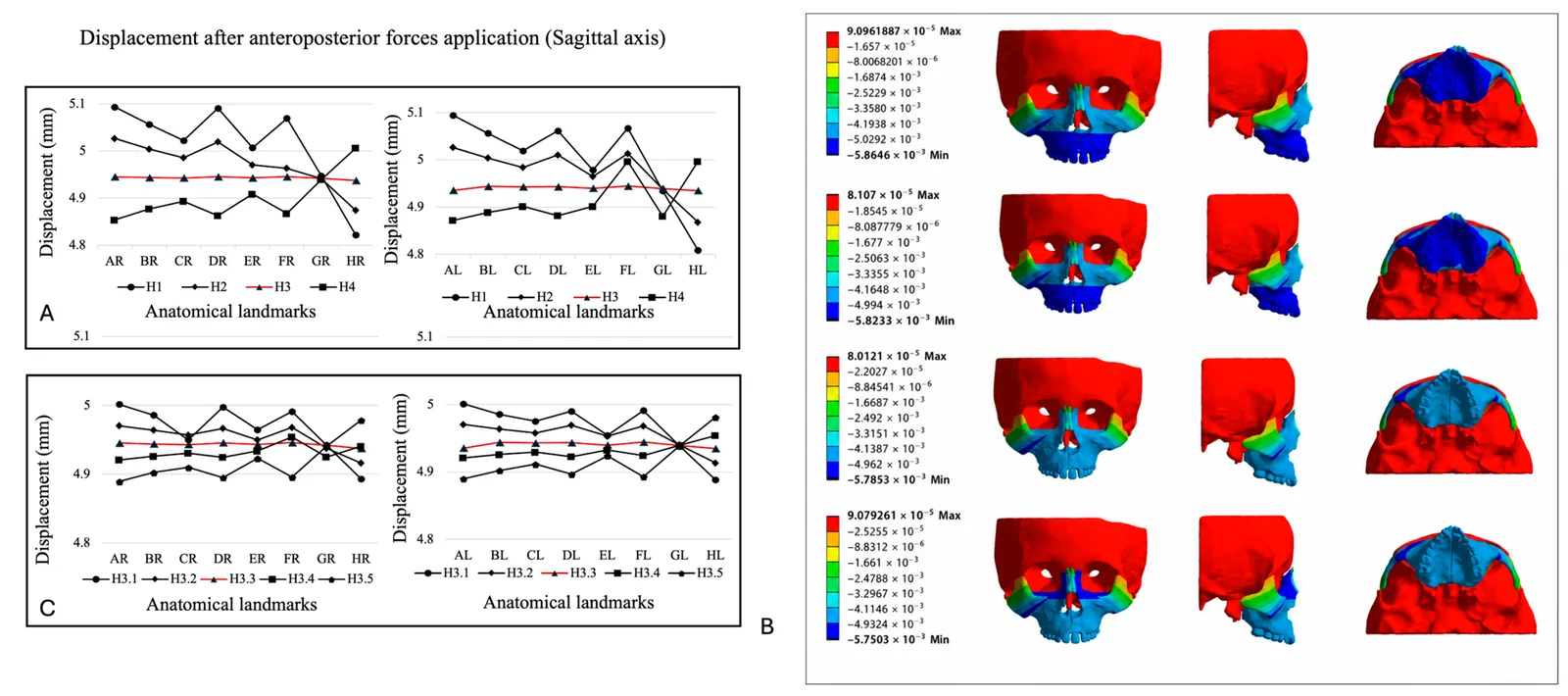
Vertical center of resistance under Anteroposterior loading: displacement (**A**), magnitude (**B**), H3.1-H3.5 level (**C**).

Analysis of anteroposterior force application along the vertical axis at positions H1-H4 on the fused palatal suture showed clear displacement patterns of the nasomaxillary complex. When force was applied at the lowest position (H1), anterior movement showed a gradient pattern. The lower anatomical landmarks (points A, B, C, D, E, and F) moved forward significantly more than the upper landmarks (points G and H), as shown in Figure 4A (Supplementary Table S2). This movement pattern indicates counterclockwise rotation of the nasomaxillary complex under H1 loading. The displacement magnitude visualization in Figure 4B supports these findings, with stronger blue colors in the lower regions showing greater forward movement compared to the upper areas.

Force application at H2 produced similar rotational effects but with less counterclockwise rotation and lower displacement. Force applied at position H3 created uniform forward movement across all eight anatomical landmarks (A–H). In contrast, H4 loading produced opposite effects to H1 and H2, with points G and H (upper nasomaxillary structure) showing maximum displacement, resulting in clockwise rotation. This different displacement is clearly seen through color mapping, where upper regions display stronger blue colors compared to lower structures.

These findings suggest that force application at position H3 created the most uniform movement across all examined structures, indicating its location through the center of resistance (Cres) of the nasomaxillary complex during anteroposterior force application.

After identifying H3 as close to the Cres, a detailed analysis was conducted to precisely locate the force that produces uniform straight-line movement. Multiple force application levels were systematically tested at set distances from the H3 reference line: H3.1 (−10 mm), H3.2 (−5 mm), H3.3 (at H3), H3.4 (+5 mm), and H3.5 (+10 mm). Analysis of movement patterns showed that lower force levels (H3.1 and H3.2) caused counter-clockwise rotation of the complex, while higher levels (H3.4 and H3.5) caused clockwise rotation. Notably, force application at position H3.3 produced uniform movement across all eight anatomical landmarks, as shown in Figure 4C.

These findings indicate that force at position H3.3 creates optimal straight-line movement, representing the precise line of action needed for pure translation of the nasomaxillary complex under forward loading conditions.

#### 3.1.2. Patent Palatal Suture Model

In the patent palatal suture model, we examined the movement of the left and right nasomaxillary complex after separation. We used the H3.3 line from the fused palatal suture model as the reference point for applying forward forces. Force was applied sideways to the mid-palatal suture, showing that the Cres moved sideways due to structural changes. We analyzed movement along two directions: sagittal and transverse axes.

**Figure 5:**
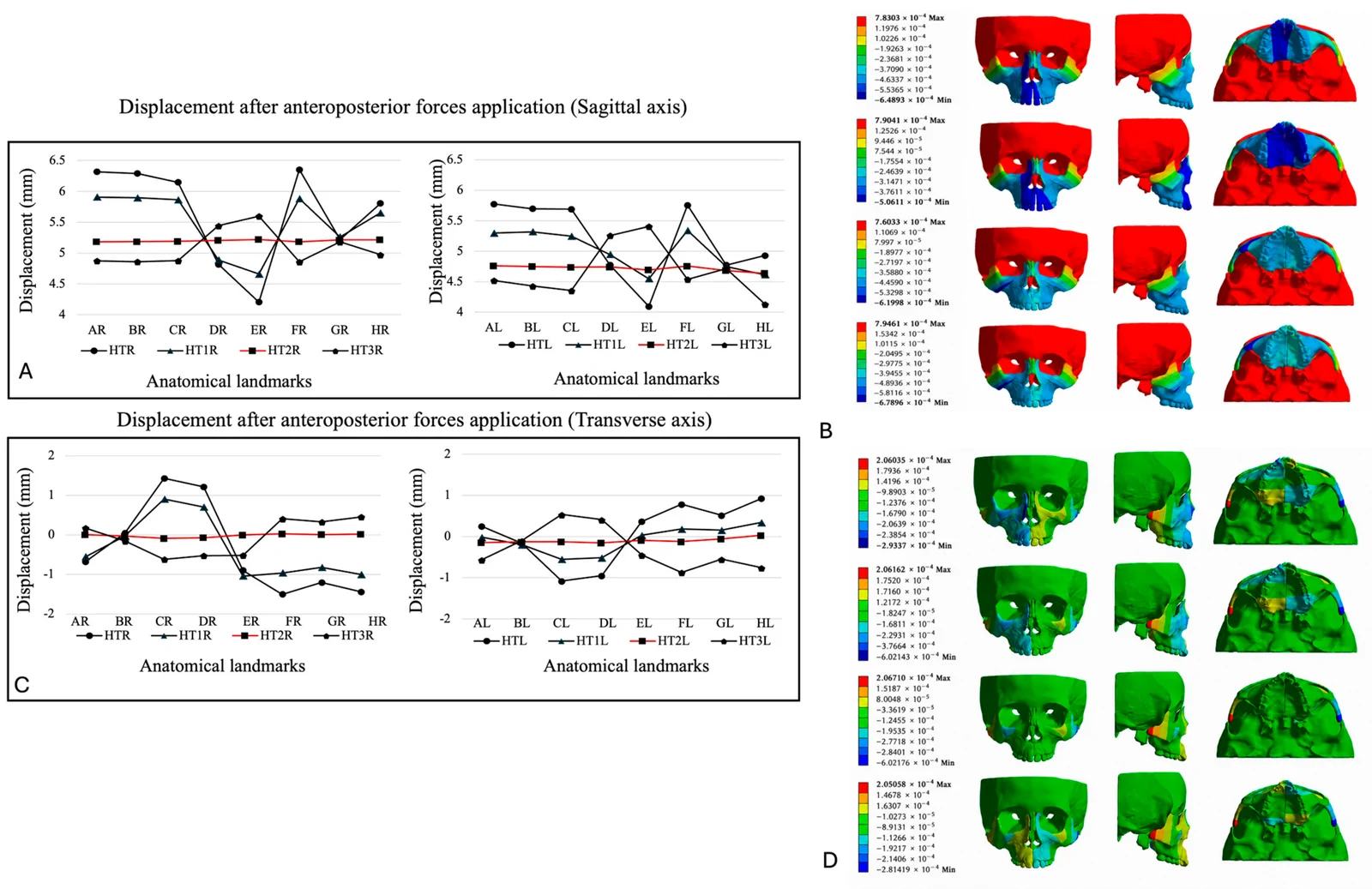
Vertical center of resistance under Anteroposterior loading, patent palatal suture: sagittal (**A**,**B**) and transverse (**C**,**D**) displacement.

Force application at the inner edge of the maxillary bone (HTR, HTL) showed distinct movement patterns. In the sagittal direction, points closer to the facial midline (A, B, C, F, and H) moved forward more than points D and E. (Figure 5A) (Supplementary Table S3). The transverse analysis showed that anterior points (A, F, G, and H) moved outward, while posterior points (C and D) moved inward, as shown in Figure 5C. Color mapping revealed important findings: sagittal analysis showed the middle area of the nasomaxillary complex had strong forward movement (Figure 5B), indicated by darker blue colors. Transverse analysis (Figure 5D) revealed expansion of the front palatal area, suggesting outward rotation of the front left and right maxilla, shown by yellow and blue colors.

Applying force 10 mm sideways from the midpalatal suture (HT1R, HT1L) produced similar but weaker rotational effects compared to the inner edge application. Notably, force application 20 mm sideways from the midpalatal suture (HT2R, HT2L) created uniform movement of the left and right maxilla, with all eight points moving forward consistently in both directions. Color mapping showed uniform blue colors in sagittal mapping and uniform green colors in transverse mapping of both maxillae.

In contrast, force application at the outer edge of the maxilla (HT3R, HT3L) caused opposite effects compared to the inner edge. The sagittal direction showed increased forward movement for points further from the midline (D and E), while the transverse direction showed more outward movement of posterior points (C and D). Color mapping suggested forward movement with expansion of the back palatal area and slight front narrowing.

In conclusion, for the patent nasomaxillary complex model, forward force application at H3.3 and 20 mm sideways from the mid-palatal suture produces the most uniform movement across all examined structures, effectively representing the Cres. All data are summarized in Table 2.

### 3.2. Determining the Sagittal Location of Center of Resistance Using Superoinferior Forces

#### 3.2.1. Fused Palatal Suture Model

**Figure 6:**
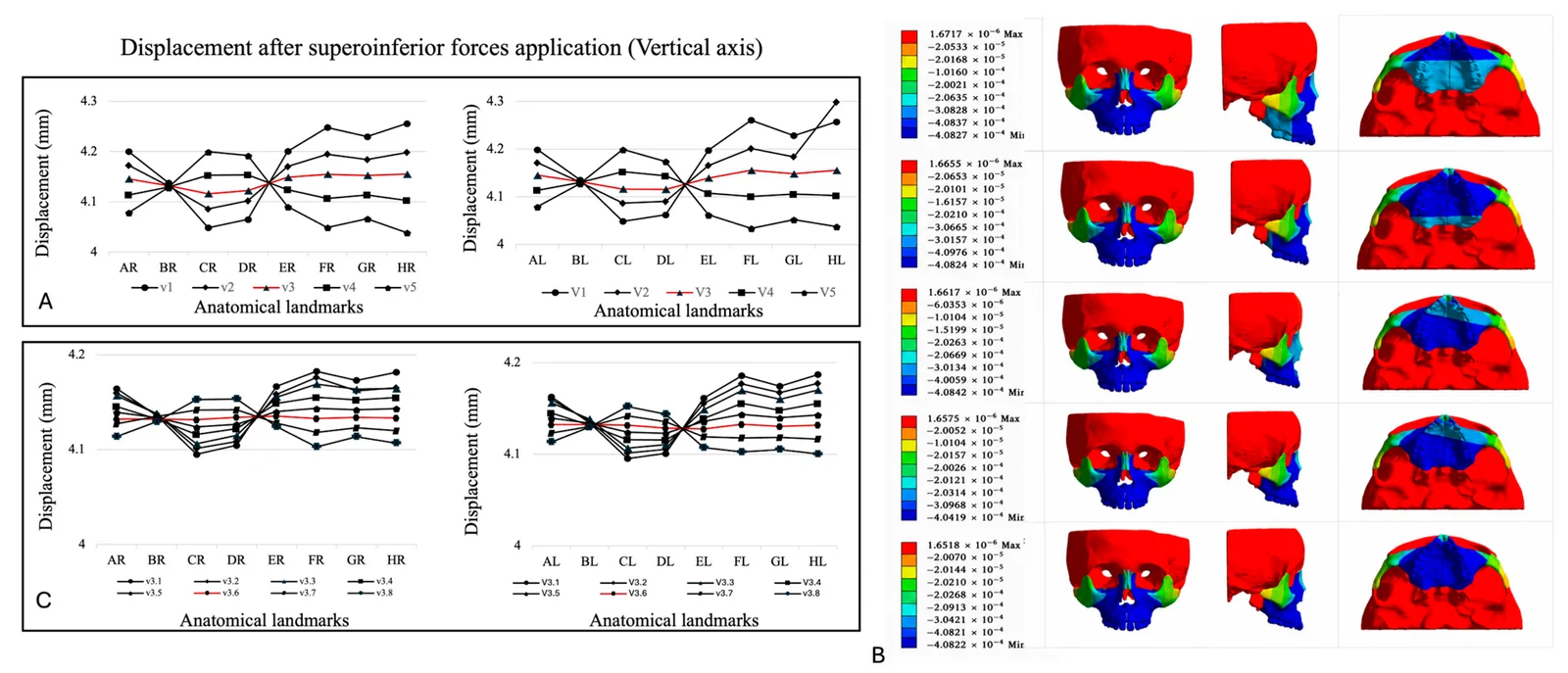
Sagittal center of resistance under superoinferior loading, fused palatal suture: displacement (**A**), magnitude (**B**), V3.1-V3.8 levels (**C**).

Analysis of superoinferior force application along the sagittal axis at positions V1–V5 showed distinct displacement patterns of the nasomaxillary complex with fused palatal sutures. Force application at position V1 showed displacement patterns, with greater downward movement in the anterior region (points A, E, F, G, H) compared to the posterior region (points C and D), resulting in clockwise rotation of the maxillary complex (Figure 6A) (Supplementary Table S4). The displacement patterns were visualized through finite element modeling, as shown in Figure 6B, where the color mapping reveals stronger blue regions in the front, showing increased downward displacement compared to the back areas of the nasomaxillary complex.

Position V2 showed similar behavior, but with less clockwise rotation and more balanced front-to-back displacement compared to V1. Position V3 demonstrated uniform downward displacement across all eight anatomical points (A–H), indicating straight downward movement of the nasomaxillary complex. In contrast, force application at V4 produced opposite effects compared to V1 and V2, with greater downward displacement of back structures relative to front landmarks, causing counterclockwise rotation. Position V5 showed similar counterclockwise rotation as V4 but with increased rotation.

These findings indicate that force application at position V3 generated an optimal translational displacement across all measured anatomical landmarks, thereby representing the Cres for superoinferior force application to the nasomaxillary complex.

Based on initial experimental results, position V3 was used as a reference point for further force analysis. A series of force application lines (V3.1–V3.8) were created in front of and behind V3 (20.0 mm), with standard 2.5 mm spacing between each line. These positions were located at distances of 27.5, 25.0, 22.5, 20.0, 17.5, 15.0, 12.5, and 10.0 mm in front of the posterior nasal spine (PNS).

Vertical force application analysis showed that positions V3.1–V3.5 produced slightly greater downward movement of front anatomical points compared to back reference points, while positions V3.7 and V3.8 showed opposite movement patterns. Position V3.6 showed uniform movement across all eight anatomical reference points, as shown in Figure 6C. These findings indicate that force application along V3.6 produced an optimal straight-line movement, representing the force line that passes through the Cres of the nasomaxillary complex vertically.

#### 3.2.2. Patent Palatal Suture Model

The movement response of the patent palatal suture model, with separated left and right maxillary segments, was measured along vertical and transverse axes. The V3.6 reference line from the fused palatal suture model was used as the standard plane for force application. Forces were directed sideways from the midline to account for the sideways-shifted Cres caused by structural changes.

**Figure 7:**
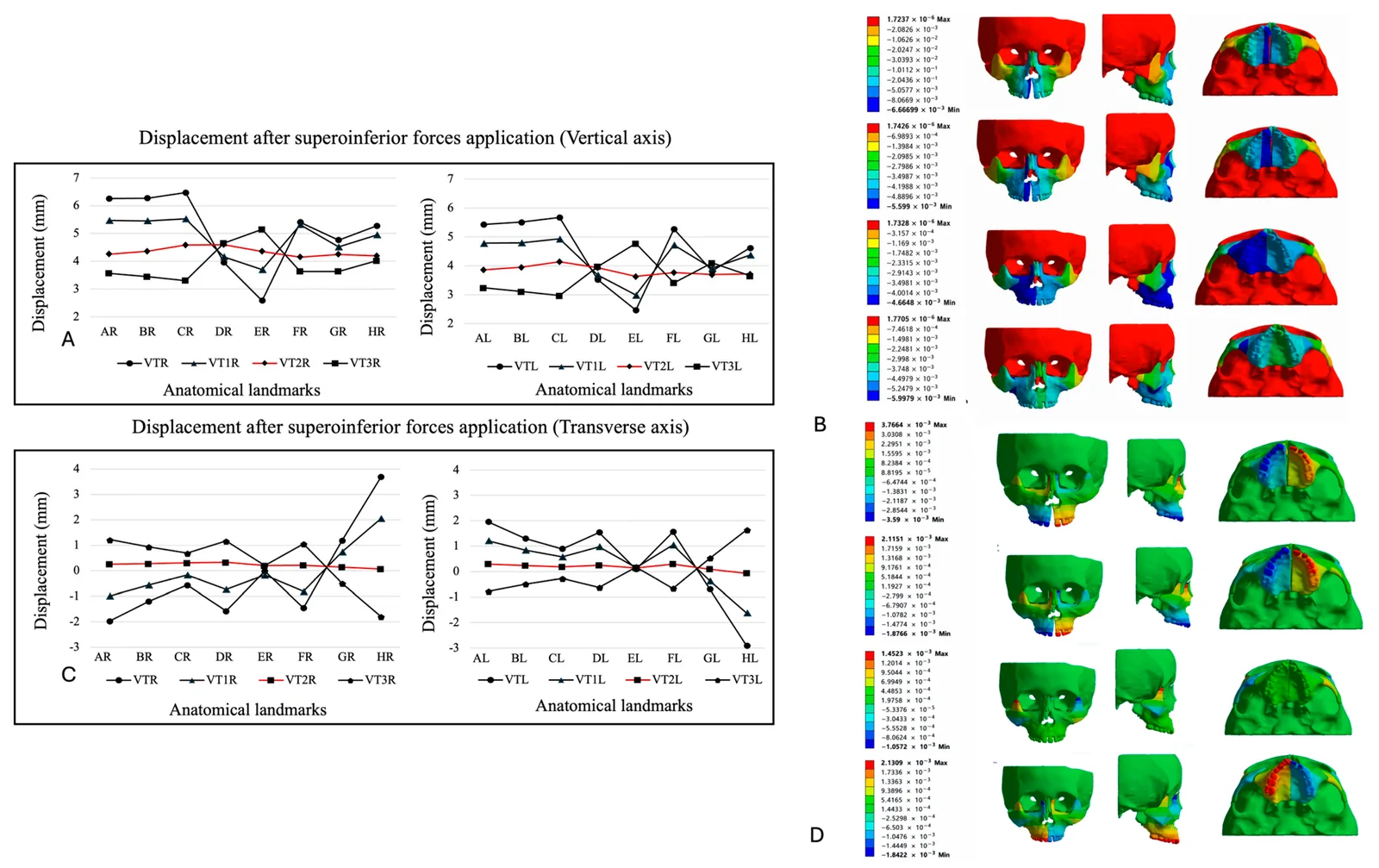
Sagittal center of resistance under superoinferior loading, patent palatal suture: vertical (**A**,**B**) and transverse (**C**,**D**) displacement.

When force was applied along the vertical axis at the inner maxillary edge (VTR, VTL), anatomical points closer to the facial midline (A, B, C, F, and H) showed significant downward movement. Point E, the most outer anatomical reference point, showed minimal vertical movement as seen in Figure 7A (Supplementary Table S5). Analysis of the transverse axis revealed different movement patterns where lower nasomaxillary complex points (A, B, C, D, F) showed strong outward expansion, while upper points (G, H) moved inward, as shown in Figure 7C. Color-coded movement mapping of the vertical axis (Figure 7B) used multiple colors to show the amount of downward movement. The inner nasomaxillary regions showed stronger blue colors compared to outer regions (shown in light green), indicating greater downward movement from inner to outer areas. Figure 7D, showing transverse axis movement, demonstrated that inner edge loading caused expansion of the lower anatomy compared to upper structures in a fan-like pattern, showing rotational spreading of the maxillary segments.

Force application at 10 mm sideways from the mid-palatal suture (VT1R, VT1L) produced similar but weaker responses compared to inner edge loading. The color visualization showed sideways spread of movement from inner nasomaxillary structures with reduced rotation. Forces applied 20 mm sideways from the mid-palatal suture (VT2R, VT2L) created mainly straight-line movement of the maxillary segments along the vertical axis without significant rotation. All anatomical points (A–H) showed uniform downward movement, appearing as uniform color distribution (dark blue on the right segment, blue on the left segment) in vertical axis mapping. Transverse axis analysis confirmed no rotational effects.

In contrast, outer edge loading (VT3R, VT3L) caused opposite responses compared to inner loading. Vertical axis analysis showed greater movement of outer point E compared to inner points. This was confirmed by color visualization showing stronger blue colors on the outer side changing to light green on the inner side. Transverse axis analysis showed slight inward rotation of the lower maxillary segments at the palatal region.

These findings indicate that force application at V3.6 and 20 mm sideways from the mid-palatal suture creates optimal uniform movement across all maxillary segments, representing the vertical force through the Cres in the patent palatal suture model. All data are summarized in Table 3.

## 4. Discussion

**Figure 8:**
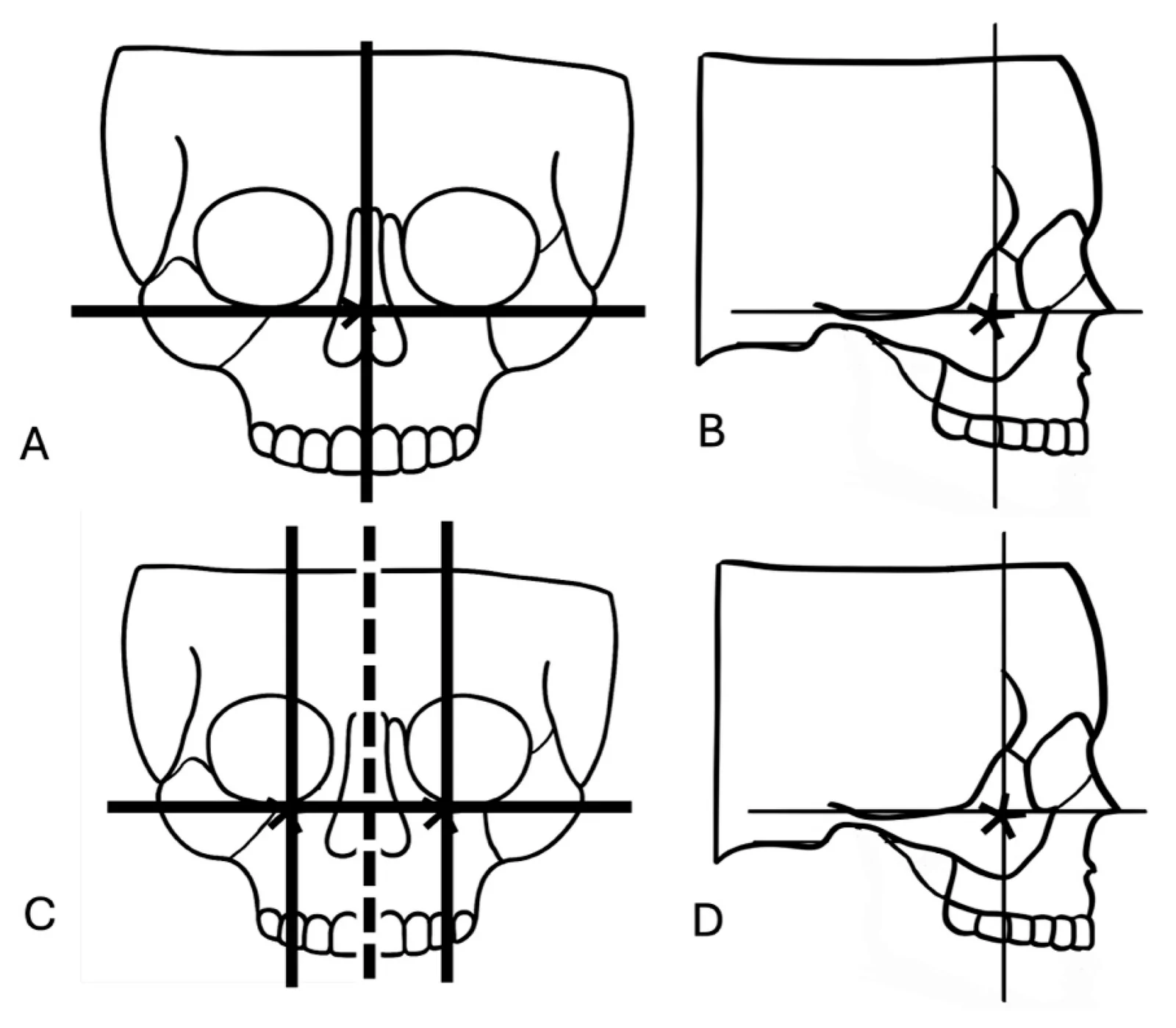
Schematic illustration of the nasomaxillary center of resistance (Cres). (**A**,**B**) Frontal and lateral views of the fused palatal-suture configuration, respectively; (**C**,**D**) frontal and lateral views of the patent palatal-suture configuration, respectively.

This study aimed to determine the mechanical behavior of nasomaxillary protraction and provide clinically relevant data to improve orthodontic appliance design and treatment protocols. Specifically, this study seeks to determine how suture opening status influences center of resistance (Cres) location, thereby informing evidence-based clinical decision-making for Class III malocclusion treatment. It is widely accepted that orthodontic or orthopedic forces should be applied relative to the Cres to achieve desired movements of the teeth and nasomaxillary complex. Finding the exact Cres in the nasomaxillary complex has been challenging due to its complex structure and limitations in measurement techniques. Importantly, this study shows that the Cres location differs between growing and non-growing patients. Understanding how the Cres differs between patients with fused versus open palatal sutures is crucial for proper force application and tooth movement. This study used finite element analysis (FEA) to precisely locate the Cres in both conditions. The study shows that the Cres of the nasomaxillary complex is located slightly behind the lower border of the malar bone under superoinferior force, and at the level of the infraorbital rim under anteroposterior force, consistent across both open and fused palatal suture conditions, as shown in Figure 8. The main difference between these conditions appears in the transverse plane, where open palatal sutures result in independent bilateral maxillary segments, requiring individual evaluation of Cres and producing sideways translation of the Cres.

The center of resistance of the nasomaxillary complex has been studied extensively through multiple methods over several decades. Various analytical techniques have been used, including two-dimensional lateral cephalometric analysis [23], laser speckle interferometry [24], photoelastic stress analysis [25], laser holography [25], and finite element analysis [14]. The present study used FEA, an advanced computational technique that allows simulation of mechanical force distribution patterns across bone and dental structures, enabling measurement of predicted displacement patterns. Within orthodontic research, this method permits detailed non-invasive examination of complex biological structures and mechanical interactions that would otherwise be challenging or impossible to investigate through conventional experimental methods, with considerable precision. Furthermore, this approach allows clinicians to virtually evaluate multiple treatment scenarios and force systems before clinical use, potentially improving treatment efficiency and minimizing complications [12,26,27].

Under the assumptions of linear elastic finite element analysis, the predicted location of the center of resistance is theoretically independent of the magnitude of the applied force. Increasing the force magnitude proportionally increases displacement but does not alter the location of the center of resistance, provided the material remains within the linear elastic range. Accordingly, the 10 N force used in this study was selected as a clinically relevant loading condition for evaluating displacement patterns and determining the location of the center of resistance.

Enlow et al. (1971) [23] initially investigated the Cres using two-dimensional lateral cephalometric analysis, concluding that the precise location remained unclear, though suggesting a position within the posterior-superior cranial region, specifically within the temporal area. The present study demonstrates that the center of resistance can be characterized along two distinct axes. Along the vertical axis, the Cres was identified at position H3.3, corresponding anatomically to the lower border of the nasal bone near the infraorbital rim. Figure 8 shows this finding, which agrees with previous research by Tanne et al [14]. However, several studies have reported different vertical positions. S. Hata et al. demonstrated that protraction forces applied 5 mm above the palatal plane generated combined parallel forward displacement with minimal forward rotation [28]. Two distinct studies located the vertical Cres at the lower margin of the zygomatic process of the maxilla (key ridge) [25,29]. Additionally, Braun et al. reported the Cres at a position halfway between the functional occlusal plane and a superior plane parallel to the functional occlusal plane intersecting the cephalometric point Orbitales [30]. The shape variations between cranial specimens and methodological differences may account for these discrepancies. When evaluating the sagittal axis, our study identified the Cres at position V3.6, corresponding to a location slightly behind the key ridge area. Three previous studies support this finding, having identified the Cres at the key ridge or back contact point of the maxillary first molar in adult skulls [25,29,30]. In contrast, Tanne et al. positioned the Cres at the posterosuperior ridge of the pterygomaxillary fissure [14].

Previous research had mainly focused on identifying the transverse Cres for balanced maxillary expansion resulting in pyramidal expansion patterns [31] or examining mechanical changes with bone-borne and tooth-borne appliances [32], yet no studies have evaluated the center of resistance within an open palatal suture model, representing a significant knowledge gap in orthopedic treatment planning. This study addresses this gap in orthopedic treatment planning. This study extends previous finite element investigations by evaluating the transverse force application required to achieve translational movement in a pediatric craniofacial model with a patent palatal suture.

An independent search across multiple vertical and horizontal levels was conducted in the patent-suture model, and this search confirmed H3.3 and V3.6 as the appropriate reference points. In the interest of manuscript conciseness, we chose to present the H3.3 and V3.6 lines as the primary reference points without including the full set of intermediate results from this independent search. We recognize, however, that this approach may have inadvertently created the impression that these values were assumed rather than independently verified.

Conventional facemask therapy typically causes forward-upward maxillary displacement with counterclockwise rotation, increasing lower facial height and potentially compromising facial appearance [18,19,33]. Our findings suggest important appliance modifications to reduce these undesirable effects. Protraction forces applied near the midline on open palatal sutures (position HT) caused bilateral maxillary separation with forward expansion and back narrowing, presenting therapeutic advantages for patients with maxillary underdevelopment, particularly those with cleft lip and palate who demonstrate transverse deficiency and front palatal narrowing [34]. On the other hand, previous reports showed front palatal narrowing when protraction appliances with reverse headgear were attached to maxillary first molars [28,35], corresponding to our findings of mesial rotation at position HT3. Therefore, when palatal expansion is not indicated, sideways force application or targeting the center of resistance represents the optimal approach, preventing unnecessary transverse changes while maintaining sagittal advancement. For fused palatal suture cases representing late adolescence or adults requiring distraction osteogenesis to correct skeletal Class III malocclusion with maxillary underdevelopment, our findings enable controlled maxillary advancement along vectors parallel to the Frankfort horizontal plane with minimal vertical changes through strategically directed forces at the identified Cres. Precise force vector control in distraction osteogenesis enables predictable outcomes, as force magnitude and direction can be customized according to individual skeletal configuration, occlusal plane orientation, and treatment objectives, transforming empirical approaches into evidence-based protocols [36,37,38]. These findings may also support future patient-specific treatment through integration with AI-assisted CBCT segmentation, computer-aided design, and 3D printing technologies. Such digital workflows could facilitate individualized appliance design and optimize force-vector application based on the predicted center of resistance [39,40].

This study used FEA to evaluate the Cres in an adolescent cranial model exhibiting maxillary underdevelopment, representative of skeletal deformity. Although FEA is well-established for assessing craniofacial displacement and stress distribution in orthodontic and orthopedic research [27,41,42], two limitations warrant discussion.

First, computational models cannot fully capture the biological variability of living tissue. In vivo validation of the Cres, while ideal, is precluded by the ethical and practical constraints of invasive procedures. Prior validation efforts using adult dry skulls or cadaveric specimens [25] offer the closest approximation to in vivo conditions but still fail to replicate the mechanical complexity of living tissue. This challenge is compounded in growing individuals, where dynamic changes in craniofacial sutures and bone properties further limit the applicability of dry skull models [23]. Given these constraints, this study relied on established FEA methodologies and material property data from the literature, representing the most rigorous non-invasive approach currently available. Additionally, all materials were modeled as homogeneous, isotropic, and linearly elastic, assumptions common in finite element research but ones that simplify the true mechanical behavior of craniofacial tissue. The model also did not distinguish between cortical and cancellous bone. Although craniofacial bone is biologically heterogeneous and anisotropic, this distinction was not incorporated into the model because craniofacial sutures, being substantially more flexible than bone, dominate deformation under orthopedic loading.

Second, the Cres is influenced by multiple variables, particularly individual craniofacial morphology, which varies with age and patient. This study used a 10-year-old model, selected because this age represents the optimal window for orthopedic treatment [43]. Future research should examine additional age groups and craniofacial anomalies, such as Crouzon, Apert, Pfeiffer, and Treacher Collins syndromes, to improve clinical applicability across diverse patient populations.

## 5. Conclusions

Within the limitations of this finite element model, palatal suture status influenced the predicted transverse location of the center of resistance. In the fused model, the estimated center of resistance was located at H3.3 vertically and V3.6 sagittally. The patent model showed similar vertical and sagittal locations; however, predominantly translatory displacement required an additional transverse offset of approximately 20 mm lateral to the midpalatal suture. These findings may provide useful biomechanical information for the design of orthopedic force systems. Further subject-specific and experimentally validated studies are required before direct clinical application.

## Figures and Tables

**Table 1:** Mechanical properties of different materials in the model.

Material	Young’s Modulus (MPa)	Poisson’s Ratio
Tooth	2.0 × 10^4^	0.3
Compact bone	1.4 × 10^4^	0.3
Suture	1.46	0.4

Note: Material properties were obtained from previously published finite element studies [16].

**Table 2:** Anteroposterior Force Application.

Model Type	Force Position	Movement Pattern	Center of Resistance Location	Biomechanical Interpretation
Fused Palatal Suture	H1 (anterior nasal spine)	Counterclockwise rotation	Below optimal level	Lower landmarks moved more than upper
	H2 (midway between occlusal/orbital)	Slight counterclockwise rotation	Below optimal level	Less rotation than H1
	H3.3	Uniform translational movement	Infraorbital rim level (15 mm superior to midway between the occlusal plane and the infraorbital rim.)	Optimal force application point
	H4 (30 mm superior to H2)	Clockwise rotation	Above optimal level	Upper landmarks moved more than lower
Patent Palatal Suture	HT (medial rim)	Bilateral separation with anterior expansion	Suboptimal	Front expansion, back constriction
	HT1 (10 mm lateral)	Reduced rotational effects	Improved but suboptimal	Less separation than HT
	HT2 (20 mm lateral)	Uniform bilateral movement	H3.3 + 20 mm lateral to midpalatal suture	Optimal for patent sutures
	HT3 (lateral rim)	No anterior expansion	Suboptimal	Back expansion, front narrowing

**Table 3:** Superoinferior Force Application.

Model Type	Force Position	Movement Pattern	Center of Resistance Location	Biomechanical Interpretation
Fused Palatal Suture	V1 (40 mm anterior to PNS)	Clockwise rotation	Anterior to optimal	Greater front downward movement
	V2 (30 mm anterior to PNS)	Slight clockwise rotation	Slightly anterior to optimal	Less rotation than V1
	V3.6	Uniform translational movement	15 mm anterior to PNS (slightly distal to zygomatic process lower border)	Optimal force application point
	V4 (10 mm anterior to PNS)	Counterclockwise rotation	Posterior to optimal	Greater back downward movement
	V5 (at PNS)	Strong counterclockwise rotation	Posterior to optimal	Maximum back movement
Patent Palatal Suture	VT (parasagittal margins)	Fan-like expansion	Suboptimal	Lower outward, upper inward movement
	VT1 (10 mm lateral)	Reduced rotational spreading	Improved but suboptimal	Less expansion than VT
	VT2 (20 mm lateral)	Uniform vertical movement	V3.6 + 20 mm lateral to midpalatal suture	Optimal for patent sutures
	VT3 (lateral rim)	Slight inward rotation	Suboptimal	Outer movement greater than inner

## Data Availability

The datasets used and/or analyzed during the current study are available from the corresponding author on reasonable request.

## Supplementary Materials

The following supporting information can be downloaded at: https://www.mdpi.com/article/10.3390/bioengineering13080903/s1, Table S1: Mesh convergence analysis showing the effect of mesh refinement on displacement at Point A. Convergence was achieved when the displacement difference between successive mesh refinements was below 1%; Table S2: Numerical displacement data corresponding to Figure 4. Total displacement magnitude (mm) of bilateral craniofacial landmarks in sagittal plane (Y-axis) under anteroposterior loading (Y-axis) at horizontal force application levels H1–H4 (A) and H3.1–H3.5 (B) in fused palatal suture model; Table S3: Numerical displacement data corresponding to Figure 5. Total displacement magnitude (mm) of bilateral craniofacial landmarks in sagittal displacement (Y-axis) (A) and transverse displacement (X-axis) (B) under anteroposterior loading at vertical force application levels H3.3 in the patent palatal suture model; Table S4: Numerical displacement data corresponding to Figure 6. Total displacement magnitude (mm) of bilateral craniofacial landmarks in vertical plane (Z-axis) under superoinferior loading (Z-axis) at horizontal force application levels V1–V5 (A) and V3.1-V3.8 (B) in fused palatal suture model; Table S5: Numerical displacement data corresponding to Figure 7. Total displacement magnitude (mm) of bilateral craniofacial landmarks in vertical plane (Z-axis) (A) and transverse displacement (X-axis) (B) under superoinferior loading at vertical force application levels V3.6 in the patent palatal suture model.
