# Supplementary material for: Center of Resistance for Maxillary Protraction in Patent and Fused Palatal-Suture Configurations: A Three-Dimensional Finite Element Study
Source: Bioengineering (Basel). 2026 Aug 10;13(8):903. doi: 10.3390/bioengineering13080903 (PMC13509258; doi:10.3390/bioengineering13080903)
Supplement: Supplementary file 1 [file bioengineering-13-00903-s001.zip › bioengineering-4430669-supplementary.pdf]

Iteration	Size of element	Number of elements	Number of nodes	Displacement (of point A)	Displacement difference (%)
1:	3 mm	71,027	131,269	5.52 mm	
2:	2 mm	256,453	450,786	5.07 mm	8.15%
3:	1.2 mm	392,885	684,704	5.00 mm	1.38%
4:	1 mm	558,043	968,156	4.95 mm	1.00%

Table S1: Mesh convergence analysis showing the effect of mesh refinement on displacement at Point A. Convergence was achieved when the displacement difference between successive mesh refinements was below 1%

A

LEFT	H1	H2	H3	H4	RIGHT	H1	H2	H3	H4
AL	5.0943	5.0263	4.9354	4.8719	AR	5.0933	5.0267	4.9454	4.8539
BL	5.0562	5.0039	4.9438	4.8888	BR	5.0567	5.0041	4.9438	4.8772
CL	5.0191	4.9839	4.9429	4.9016	CR	5.0222	4.9857	4.9429	4.8935
DL	5.0614	5.0103	4.9433	4.8819	DR	5.0908	5.0194	4.9456	4.8628
EL	4.9784	4.9646	4.9397	4.9016	ER	5.007	4.9706	4.9431	4.9088
FL	5.0669	5.0136	4.9443	4.9967	FR	5.0694	4.9635	4.9457	4.8677
GL	4.9342	4.9379	4.939	4.8805	GR	4.9476	4.9416	4.9421	4.9397
HL	4.8084	4.8678	4.9347	4.9967	HR	4.8219	4.8741	4.9374	5.0064

B

LEFT	H3.1	H3.2	H3.3	H3.4	H3.5	RIGHT	H3.1	H3.2	H3.3	H3.4	H3.5
AL	5.001	4.9705	4.9354	4.921	4.8901	AR	5.0015	4.9704	4.9454	4.9208	4.8895
BL	4.9855	4.9636	4.9438	4.926	4.9022	BR	4.9856	4.9639	4.9438	4.926	4.9027
CL	4.9752	4.9577	4.9429	4.9294	4.9116	CR	4.9498	4.9569	4.9429	4.9305	4.91
DL	4.99	4.9694	4.9433	4.9228	4.8969	DR	4.9974	4.9665	4.9456	4.9247	4.8951
EL	4.9542	4.9529	4.9397	4.9326	4.9241	ER	4.9645	4.9498	4.9431	4.9338	4.923
FL	4.9912	4.9682	4.9443	4.9245	4.8931	FR	4.9909	4.968	4.9457	4.954	4.8959
GL	4.9389	4.9402	4.939	4.9396	4.9387	GR	4.9421	4.9376	4.9421	4.925	4.9406
HL	4.8884	4.9133	4.9347	4.9542	4.9814	HR	4.893	4.916	4.9374	4.9407	4.9787

Table S2: Numerical displacement data corresponding to Figure 4. Total displacement magnitude (mm) of bilateral craniofacial landmarks in sagittal plane (Y-axis) under anteroposterior loading (Y-axis) at horizontal force application levels H1–H4 (A) and H3.1–H3.5 (B) in fused palatal suture model

A

LEFT	HTL	HT1L	HT2L	HT3L	RIGHT	HTR	HT1R	HT2R	HT3R
AL	5.7724	5.296	4.7614	4.52	AR	6.3185	5.9081	5.1836	4.8743
BL	5.6962	5.3189	4.7487	4.4284	BR	6.2909	5.8963	5.1873	4.8617
CL	5.6892	5.246	4.7362	4.3544	CR	6.1476	5.8647	5.191	4.8771
DL	4.7708	4.9444	4.7408	5.2581	DR	4.818	4.8898	5.2075	5.4454
EL	4.0929	4.5467	4.6926	5.407	ER	4.2071	4.6601	5.2221	5.5999
FL	5.7555	5.339	4.7566	4.5376	FR	6.3538	5.882	5.1836	4.8584
GL	4.7728	4.7494	4.6841	4.7165	GR	5.2189	5.2617	5.2131	5.1808
HL	4.9283	4.6131	4.6363	4.1294	HR	5.8069	5.6522	5.2163	4.9732

B

LEFT	HTL	HT1L	HT2L	HT3L	RIGHT	HTR	HT1R	HT2R	HT3R
AL	0.2459	-0.017359	-0.14804	-0.56614	AR	-0.68	-0.55424	0.002272	0.17211
BL	-0.16544	-0.19827	-0.13044	-0.10033	BR	0.04138	-0.031998	-0.034039	-0.15553
CL	-1.083	-0.55288	-0.12731	0.53424	CR	1.4275	0.9028	-0.089648	-0.61971
DL	-0.95009	-0.51454	-0.15249	0.40948	DR	1.2127	0.70074	-0.071996	-0.52696
EL	0.36567	0.031348	-0.09011	-0.4492	ER	-0.89855	-1.0421	-0.0060471	-0.5188
FL	0.78388	0.18328	-0.12243	-0.87083	FR	-1.5046	-0.96392	0.024796	0.40494
GL	0.51387	0.15538	-0.056018	-0.54915	GR	-1.2067	-0.82483	0.0081034	0.32796
HL	0.92524	0.34073	0.028673	-0.75694	HR	-1.4409	-1.0058	0.016078	0.45446

Table S3: Numerical displacement data corresponding to Figure 5. Total displacement magnitude (mm) of bilateral craniofacial landmarks in sagittal displacement (Y-axis) (A) and transverse displacement (X-axis) (B) under anteroposterior loading at vertical force application levels H3.3 in the patent palatal suture model.

A

LEFT	V1	V2	V3	V4	V5	RIGHT	V1	V2	V3	V4	V5
AL	4.1984	4.171	4.1449	4.1133	4.0791	AR	4.1999	4.1724	4.1454	4.1138	4.0784
BL	4.1349	4.1302	4.1317	4.1295	4.1275	BR	4.1376	4.1312	4.1323	4.1293	4.1285
CL	4.0483	4.0864	4.1159	4.152	4.1992	CR	4.0482	4.0856	4.116	4.1527	4.1997
DL	4.0619	4.0899	4.1153	4.1436	4.1738	DR	4.0647	4.1017	4.122	4.1535	4.1922
EL	4.1972	4.1653	4.139	4.1071	4.0618	ER	4.2005	4.1702	4.1488	4.1241	4.0904
FL	4.2603	4.2009	4.1553	4.1002	4.0335	FR	4.2478	4.1947	4.1548	4.1068	4.049
GL	4.2279	4.1834	4.1479	4.1051	4.0519	GR	4.2296	4.1839	4.1523	4.1136	4.0663
HL	4.2569	4.2983	4.1553	4.1023	4.0372	HR	4.2558	4.1982	4.1551	4.1028	4.0386

LEFT	V3.1	V3.2	V3.3	V3.4	V3.5	V3.6	v3.7	v3.8	RIGHT	v3.1	v3.2	v3.3	v3.4	v3.5	v3.6	v3.7	v3.8
AL	4.1625	4.1596	4.156	4.1449	4.1391	4.1322	4.123	4.1133	AR	4.1643	4.1595	4.1568	4.1454	4.1393	4.1322	4.1272	4.1138
BL	4.1345	4.1364	4.1384	4.1317	4.1337	4.1323	4.13	4.1295	BR	4.1358	4.1377	4.1378	4.1323	4.1338	4.1323	4.1348	4.1293
CL	4.0953	4.1013	4.1066	4.1159	4.124	4.1315	4.1417	4.152	CR	4.095	4.1011	4.106	4.116	4.1239	4.1315	4.1418	4.1527
DL	4.1008	4.1054	4.1104	4.1153	4.1229	4.1283	4.1355	4.1436	DR	4.1045	4.1085	4.1149	4.122	4.1268	4.1341	4.1419	4.1535
EL	4.1612	4.1554	4.1485	4.139	4.1356	4.1275	4.119	4.1071	ER	4.1667	4.1583	4.1552	4.1488	4.1401	4.1353	4.128	4.1241
FL	4.1857	4.1768	4.1694	4.1553	4.1435	4.1325	4.1177	4.1023	FR	4.1825	4.1761	4.169	4.1551	4.1435	4.1327	4.1182	4.1028
GL	4.1742	4.1672	4.16	4.1479	4.1398	4.1302	4.1182	4.1051	GR	4.1731	4.1619	4.1637	4.1523	4.1421	4.134	4.1229	4.1136
HL	4.1869	4.1772	4.17	4.1553	4.1428	4.1317	4.1164	4.1002	HR	4.1816	4.1653	4.1643	4.1548	4.1433	4.1334	4.1199	4.1068

B

Table S4: Numerical displacement data corresponding to Figure 6. Total displacement magnitude (mm) of bilateral craniofacial landmarks in vertical plane (Z-axis) under superoinferior loading (Z-axis) at horizontal force application levels V1-V5 (A) and V3.1-V3.8 (B) in fused palatal suture model

LEFT	VTL	VT1L	VT2L	VT3L	RIGHT	VTR	VT1R	VT2R	VT3R
AL	5.435	4.7821	3.861	3.239	AR	6.2565	5.47072	4.259	3.5702
BL	5.5139	4.7959	3.9485	3.1076	BR	6.2684	5.4551	4.3588	3.4469
CL	5.6745	4.9332	4.1409	2.9706	CR	6.4673	5.527	4.5838	3.3055
DL	3.5253	3.6855	3.9377	3.972	DR	3.96	4.1598	4.5991	4.6538
EL	2.467	2.9868	3.6312	4.7731	ER	2.5886	3.6984	4.3555	5.1485
FL	5.2753	4.7214	3.7697	3.4126	FR	5.4063	5.318	4.1503	3.6385
GL	3.8203	3.9185	3.6968	4.0984	GR	4.7623	4.518	4.249	3.6385
HL	4.6232	4.3748	3.7155	3.65	HR	5.2741	4.95	4.1946	4.0204

A

LEFT	VTL	VT1L	VT2L	VT3L	RIGHT	VTR	VT1R	VT2R	VT3R
AL	1.9564	1.2114	0.29744	-0.7763	AR	-1.9678	-0.98283	0.25927	1.2253
BL	1.3005	0.84176	0.23772	-0.491	BR	-1.1981	-0.55634	0.27488	0.94092
CL	0.89192	0.57743	0.18371	-0.27588	CR	-0.56317	-0.16242	0.31179	0.69388
DL	1.5422	0.97685	0.24681	-0.62753	DR	-1.5739	-0.73003	0.33292	1.1728
EL	0.093224	0.1511	0.14733	0.17782	ER	-0.016517	-0.16866	0.2033	0.20629
FL	1.5614	1.0455	0.2966	-0.66328	FR	-1.4527	-0.8091	0.22027	1.0604
GL	-0.69363	-0.37073	0.09342	0.52917	GR	1.1941	0.74302	0.14569	-0.49472
HL	-2.9091	-1.6221	-0.069007	1.6421	HR	3.6965	2.0596	0.069212	-1.7929

B

Table S5: Numerical displacement data corresponding to Figure 7. Total displacement magnitude (mm) of bilateral craniofacial landmarks in vertical plane (Z-axis) (A) and transverse displacement (X-axis) (B) under superoinferior loading at vertical force application levels V3.6 in the patent palatal suture model.
